# Estimating cellular parameters through optimization procedures: elementary principles and applications

**DOI:** 10.3389/fphys.2015.00060

**Published:** 2015-03-03

**Authors:** Akatsuki Kimura, Antonio Celani, Hiromichi Nagao, Timothy Stasevich, Kazuyuki Nakamura

**Affiliations:** ^1^Cell Architecture Laboratory, National Institute of GeneticsMishima, Japan; ^2^Department of Genetics, School of Life Science, SOKENDAI (The Graduate University for Advanced Studies)Mishima, Japan; ^3^Transdisciplinary Research Integration Center and Data Centric Science Research Commons, Research Organization of Information and SystemsTokyo, Japan; ^4^Quantitative Life Sciences Unit, The Abdus Salam International Centre for Theoretical PhysicsTrieste, Italy; ^5^Research and Development Center for Data Assimilation, The Institute of Statistical MathematicsTokyo, Japan; ^6^Research Center for Large-Scale Earthquake, Tsunami and Disaster, Earthquake Research Institute, The University of TokyoTokyo, Japan; ^7^Department of Biochemistry and Molecular Biology, Colorado State UniversityFort Collins, CO, USA; ^8^Department of Mathematical Sciences Based on Modeling and Analysis, School of Interdisciplinary Mathematical Sciences, Meiji UniversityTokyo, Japan

**Keywords:** quantitative modeling, parameter optimization, model selection, likelihood, probability density function

## Abstract

Construction of quantitative models is a primary goal of quantitative biology, which aims to understand cellular and organismal phenomena in a quantitative manner. In this article, we introduce optimization procedures to search for parameters in a quantitative model that can reproduce experimental data. The aim of optimization is to minimize the sum of squared errors (SSE) in a prediction or to maximize likelihood. A (local) maximum of likelihood or (local) minimum of the SSE can efficiently be identified using gradient approaches. Addition of a stochastic process enables us to identify the global maximum/minimum without becoming trapped in local maxima/minima. Sampling approaches take advantage of increasing computational power to test numerous sets of parameters in order to determine the optimum set. By combining Bayesian inference with gradient or sampling approaches, we can estimate both the optimum parameters and the form of the likelihood function related to the parameters. Finally, we introduce four examples of research that utilize parameter optimization to obtain biological insights from quantified data: transcriptional regulation, bacterial chemotaxis, morphogenesis, and cell cycle regulation. With practical knowledge of parameter optimization, cell and developmental biologists can develop realistic models that reproduce their observations and thus, obtain mechanistic insights into phenomena of interest.

## Introduction: regression analyses for identifying parameter values by applying experimental data to a quantitative model

The purpose of quantitative biology is to achieve biological discovery through quantitative data analyses and modeling. A quantitative model consists of a set of rules, often expressed by mathematical formulas, which involve a set of parameters governing variables for the rules and initial/boundary conditions. The simplest way to validate a given quantitative model is to test whether an appropriate set of rules and parameters reproduces experimental observations. If it does this successfully, it can be concluded that the model (i.e., the rules and parameter values) is “sufficient” to explain the observations. However, in many cases, we do not have information on the “appropriate parameters.” In such cases, we may want to identify a set of parameters that adequately explains the experimental observations under the stated rules. If the rules adequately represent the true mechanisms underlying the biological process, the identified parameters should reflect the quantitative properties of that process. In this way, we can argue that the model (i.e., the rules and the “estimated”parameter values) is sufficient to explain the observations. The method for estimating parameters by fitting a given quantitative model to the observed data is called regression, and the overall workflow is comprehensively reviewed in Jaqaman and Danuser (2006). In this article, we focus on several practical procedures for identification of parameters and introduce recent applications of regression for characterization of cellular processes.

## Sum of squared errors (SSE) of prediction and likelihood as indices of parameter optimization

Minimization of the SSE and maximization of likelihood (abbreviated as “LS” and “ML,” respectively, in Jaqaman and Danuser, 2006) are the two most common regression schemes. We first review SSE and likelihood before explaining the methods for minimizing/maximizing these indices in Sections Minimization of SSE and Maximization of Likelihood. Minimization of SSE has been widely used as a simple and straightforward method to obtain an optimum parameter set. However, SSE does not provide further information, such as the uncertainty of the determined parameter values. In contrast, likelihood, which is a powerful concept that covers the shortcomings of SSE, is capable of estimating both an optimum parameter set and a probability density function (PDF) related to the parameters, taking experimental error and the imperfections of the model into account. We sometimes encounter a problem in selecting an optimum model from among candidate models that contain different numbers of parameters. In Section Model selection Using Likelihood, we introduce information criteria, which enable us to solve this problem when used in combination with likelihood.

### Minimization of SSE

**Figure 1:**
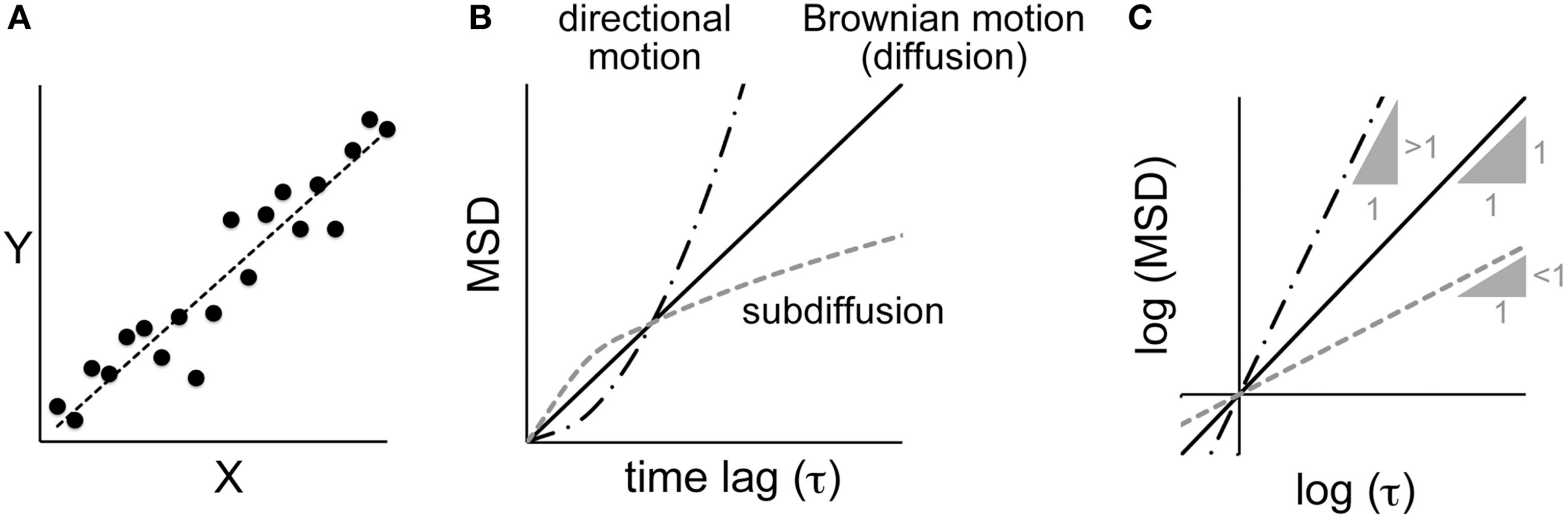
**Correlation analyses between parameters**. (**A**) A linear correlation and linear regression. *X* and *Y* are two parameters of a dataset. Plotting the values of *Y* against *X* shows a correlation between the parameters, and the extent of that correlation can be calculated by regression analysis. (**B**) The relationship between the mean square displacement (MSD) and the time lag for various modes of motion (see text for details). (**C**) The same plot as shown in (**B**), except using logarithmic values. The three lines correspond to the different modes of motion in (**B**). For Brownian motion, the slope of the log–log plot is one. For directional motion and sub-diffusion, the log–log plots yield a linear relationship with a slope greater than one and less than one, respectively.

Linear regression is the most familiar example of regression (Bremer and Doerge, 2010). When an obvious linear correlation is identified between two variables through a regression analysis (e.g., *X* and *Y* in Figure 1A), we can assume a model, formulated as *Y* = *a*_0_ + *a*_1_ *X*, that describes the relationship between the variables. To identify the parameters of the model (i.e., *a*_0_ and *a*_1_) that reproduce the experimental observations, a least-square method is frequently used (Bremer and Doerge, 2010). In this method, we define an evaluation function that sums the squared distance between the experimental data and the model with a given set of parameters. The SSE, which is defined as SSE = Σ_*i*=1_*^n^* [*y_i_*–(*a*_0_ + *a*_1_ *x_i_*)]^2^, where *n* is the number of data points and (*x_i_*, *y_i_*)(*i* = 1, …, *n*) are the data, is commonly used as an index for the least-squares method. Parameters that minimize the evaluation function are the optimum parameters, in the sense that they minimize the discrepancy between the model and the experimental results.

As a biological example of linear regression, we have demonstrated that there is a correlation between the cell size and the extent and speed of the elongation of the mitotic spindle in *Caenorhabditis elegans* embryos (Hara and Kimura, 2009). In this study, we further demonstrated that the elongation of the mitotic spindle depends on cell size by showing that the elongation of the mitotic spindle increased when we increased the cell size.

As another example, let us consider the motion of a particle inside a cell (Figure 1B). If the motion is driven by random Brownian forces, the mean square displacement (MSD) is linearly proportional to the time lag (τ) (i.e., MSD ∝ τ^1^) (Berg, 1993). The motion of a particle inside a cell is rarely random because it is confined to a crowded space. The MSD decreases, and such motion is called “sub-diffusion” (i.e., MSD ∝ τ^α^,α < 1) (Saxton and Jacobson, 1997). In other cases, the particle may be moved by directional flow, and thus will be moved further than it would by random diffusion (i.e., MSD ∝ τ^α^,α > 1). If we could estimate the value of α in the MSD-vs.-τ plot, we would be able to determine whether the motion is better explained by random Brownian diffusion, sub-diffusion, or directed flow. To estimate α, a log–log plot is useful (Figure 1C). In the log–log plot, i.e., (log MSD) = α(log τ) + (log *C*), α is the slope and (log *C*) is the intercept of the line. Therefore, using the above-mentioned linear regression analysis, we can identify the value of α that minimizes SSE in the (log MSD)-vs.-(log τ) plot.

Such linear regression analysis of a double logarithmic plot is useful in characterizing how cellular parameters affect each other. We quantified the shape of mitotic spindles in *C. elegans* embryos and found a relationship described by *SW* = 1.5 × *P*^0.36^ × *HL*^0.58^, where *SW* and *HL* are the width and hypotenuse length of the spindles and *P* is the ploidy of the embryos. Based on this formulation, we were able to propose a physical model that explains spindle shape (Hara and Kimura, 2013).

Minimization of SSE is applicable to both linear relationships and a variety of estimations. Because SSE is defined as the sum of the squared difference between the value estimated using the model and the actual observations, the value can be defined for any type of quantitative model. For example, in fluorescence recovery after photobleaching (FRAP) experiments, the recovery curve for the fluorescence intensity of the region where the fluorescent molecules were bleached can be modeled as an exponential curve, with its gradient reflecting the diffusion constant of the molecule (Axelrod et al., 1976). By identifying the parameter that minimizes the SSE between exponential curves and the experimental data for fluorescence intensity, one can estimate the diffusion constant of the molecule.

### Maximization of likelihood

The simplicity of the SSE, which is a straightforward index for the discrepancy between a given model and the observations, sometimes causes difficulties in real data analyses. Suppose that, for example, a phenomenon of interest is characterized by parameters having different physical dimensions (e.g., length and weight). How can we compute the sum of errors in different dimensions? In such a case, the observational data should be converted to dimensionless quantities through standardization of each type of data. Likelihood is another important index to evaluate how well a given model agrees with experimental results. Since the definition of likelihood naturally converts the observational data into dimensionless data, usage of likelihood can, unlike SSE, avoid the difficulty mentioned above. One of the major advantages of likelihood over SSE is that we can obtain both an optimum parameter set and a PDF related to the parameters. The obtained PDF provides valuable information not only of an optimum value for each parameter but also of its uncertainty due to errors contained in the observational data and the imperfections of the given model.

**Figure 2:**
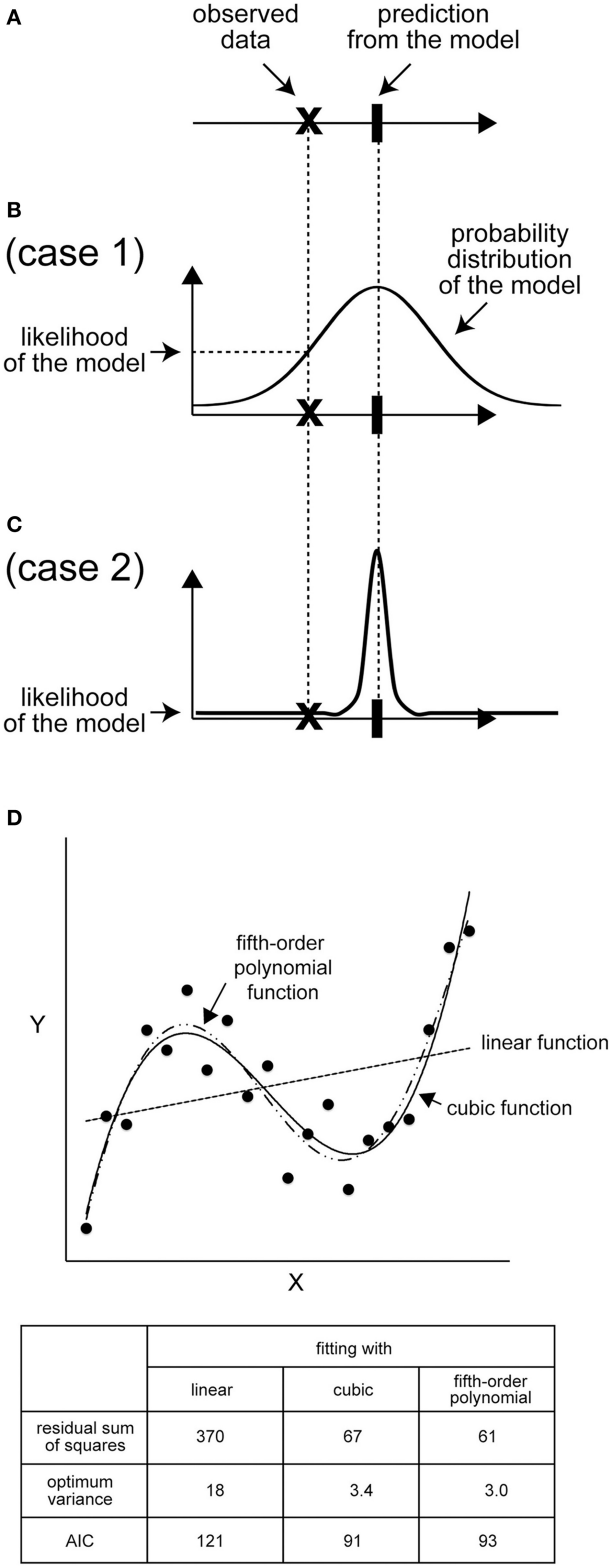
**Likelihood: the distribution is important. (A)** An example of the mean of predicted values and observed data points. **(B)** If the distribution of the predicted values of the model is broad, the likelihood of the model is high because the probability of observing the data is high. **(C)** In contrast, if the distribution of the predicted value is narrow, the likelihood will be low. **(D)** An example of AIC calculation. Black dots represent an imaginary set of observed data. For *x* = 1, 2, 3, …, 20, the *y* value was calculated according to *y* = 0.025 × (*x* - 3) (*x*-10) (*x* − 17) + 10, and a Gaussian noise correction with a variance of four was added to each *y* value. Next, we calculated the best-fit linear, cubic, and fifth-order polynomial functions for the 20 data points. *l*_max_ = −(*n*/2) × ln(2πσ^2^) – (1/2σ^2^) × Σ_*i*=1_*^n^* [*y_i_* - *y*_model_(*x_i_*)]^2^, where *n* is the number of data points (*n* = 20), σ^2^ is the variance of the model, and *y_i_* and *y*_model_(*x_i_*) are observed and model values, respectively, at *x* = *x_i_*. The sum of squared residuals is Σ_*i*=1_*^n^*[*y_i_* - *y*_model_(*x_i_*)]^2^. AIC is calculated as AIC = 2*k* − 2*l*_max_, where *k* is the number of free parameters in the model and is 3, 5, and 7 for linear, cubic, and fifth-order functions, respectively. Note that the variance of each model is also a free parameter to be optimized.

Let us consider a situation in which 1.1 is the experimental value (*x*), while a given model predicts that *x* should be 1.0 (Figure 2A). How good is this model? (In other words, how “likely” is this model to describe the experimental result?) A conditional PDF related to an experimental value when results of the model are given is required to calculate the likelihood; a single value, such as a mean value, is insufficient. Suppose that we conduct simulations many times, and obtain results that follow a normal distribution with a mean (μ) of 1.0 (σ) of 1.0. The likelihood (*L*) indicates, roughly, the probability that the model yields the experimental value. For our current example, the likelihood is *L* = (2πσ^2^)^−1/2^ exp[−(*x* − μ)^2^/2σ^2^] = 0.4, where the experimental value is 1.1 (Bishop, 2006; Kitagawa, 2010). If we had independently observed multiple experimental data points {*x*_1_, *x*_2_, …, *x_n_*} for *x*, the likelihood of the dataset is given as a product of the likelihood of each data point, i.e., *L* = Π_*i* = 1_*^n^L_i_*. Often, we use log-likelihood, *l* = ln *L*; thus, the total log-likelihood of the model can be shown as *l* = ln (Π_*i*=1_^*n*^L_*i*_) = Σ_*i*=1_^*n*^ *l_i_*. The likelihood *L*, or the log-likelihood *l*, is originally an indicator of how likely the obtained experimental data are, based on a model with a given parameter set. The larger the likelihood or log-likelihood, the better the model reproduces the observation. In the example shown in Figure 2, even when the distance between the observation and the mode of each likelihood function, i.e., the best observation that attains the likelihood function maximum, is equal for candidate models, we can reasonably select a model that has a broad likelihood function as the better model (Figures 2B,C). In turn, the parameter set that maximizes *L* or *l* is considered to be optimum to explain the experimental data. This method for estimating the optimum parameters is called the “maximum likelihood method.”

### Model selection using likelihood

When we wish to evaluate the validity of a model, a straightforward approach is to test whether the model can predict unknown data sets. Cross-validation and bootstrap methods are examples of such strategies (Hastie et al., 2009). As another strategy, we can select good models using likelihood as the index, just as we select good parameter values using likelihood. For example, suppose that the growth rate of a cell is found to increase when gene *X* is mutated, and that a theoretical framework that explains the growth rate of wild-type cells exists. The model selection procedure enables us to determine a better model among candidates: model 1, gene *X* affects one parameter (e.g., protein production rate); or model 2, gene *X* affects two parameters (e.g., protein production rate and RNA production rate).

We often have to consider selecting the best model among models that contain different numbers of parameters. In general, a model that contains more parameters tends to attain larger likelihood since it easily fits to observed data. However, the use of too many parameters leads to overfitting, in which the model loses predictability despite fitting well to observations.

To select a model that fits well to observed data and minimizes the number of parameters to avoid overfitting, the Akaike information criterion (AIC) is widely accepted in various fields of science (Akaike, 1974). The AIC is theoretically derived to be AIC = −2*l*_max_ + 2*k*, where *k* is the number of free parameters in the model and *l*_max_ is the maximum log-likelihood. The model with the smallest AIC is selected as the best one. The Bayesian information criterion (BIC) is another index used for model selection. BIC is slightly different from the AIC in the additional term, which penalizes the number of parameters more severely than the AIC (Jaqaman and Danuser, 2006). Example of the use of both AIC and BIC can be found in modeling of a FRAP experiment (Darzacq et al., 2007) and in identifying low-dimensional models to reproduce cell cycle regulations (Kondo et al., 2013).

Figure 2D shows an example of model selection using the AIC. The data are synthetically generated from a cubic function, *y* = 0.025 × (*x* − 3) (*x* − 10) (*x* − 17) + 10 + ε, where ε is the observational noise, which follows a normal distribution with a mean of zero and a variance of four. We give candidate models for comparison with the observed data as a linear function (*y* = θ_1_ + θ_2_*x* + ε_1_), a cubic function (*y* = θ_1_ + θ_2_*x* + θ_3_*x*^2^ + θ_4_*x*^3^ + ε_2_), or a fifth-order polynomial function (*y* = θ_1_ + θ_2_*x* + θ_3_*x*^2^ + θ_4_*x*^3^ + θ_5_*x*^4^ + θ_6_*x*^5^ + ε_3_), where ε_1_, ε_2_, and ε_3_ are Gaussian noises. Under this assumption, the optimum parameter set (θ_i_) determined based on the maximum likelihood method coincides with the solution of the least-squares method (Bishop, 2006). The sum of squared residuals is the smallest in the case of the fifth-order polynomial function, as expected, because the function contains more free parameters than the other models (Figure 2D). In contrast, the AIC is the smallest in the case of the cubic function owing to the penalty term that inhibits a needless increase in the number of parameters (Figure 2D). Therefore, the AIC successfully selects the true cubic function as the best model avoiding the over- or under-parameterized models.

## Procedures to optimize parameters

**Figure 3:**
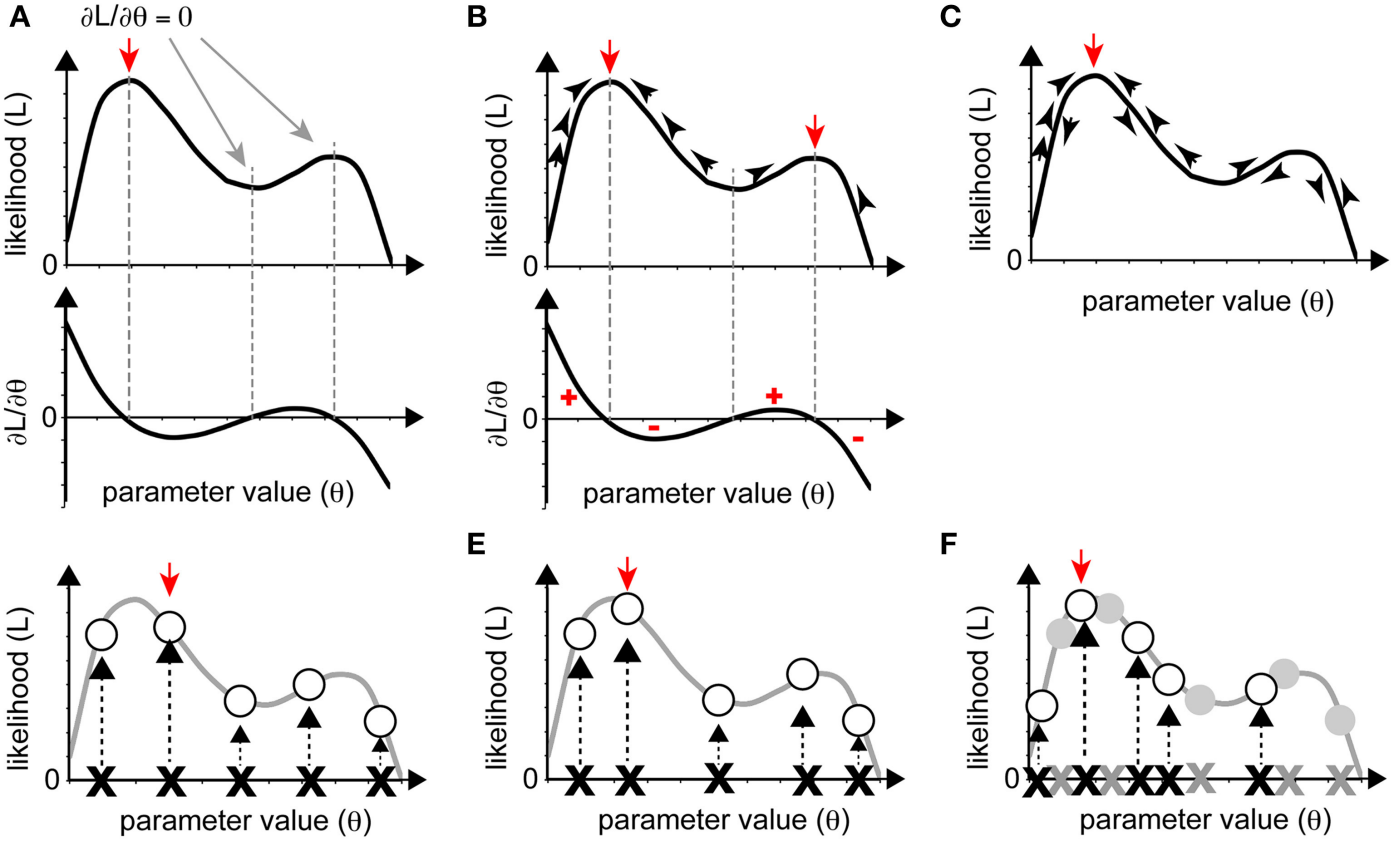
**Various optimization strategies. (A–C)** Gradient approaches. **(A)** When the partial differential equations for likelihood can be solved as functions of parameters, the solutions yield local maxima or minima (red and gray arrows). The red arrow indicates maximum likelihood. **(B)** We can reach local maxima (red arrows) by iteratively following the gradient from a starting point. **(C)** If, in following the gradient, we add stochasticity, we may avoid being trapped in a local maximum and reach the global maximum (red arrow). **(D–F)** Sampling approaches. The red arrow indicates the sampling point with the highest likelihood. **(D)** Grid sampling, in which sampling occurs at regular intervals. **(E)** Simple random sampling, where parameters are chosen at random. **(F)** Importance sampling was added to **(E)**. In the second round of sampling, more realizations were set near the realization with high likelihood from the initial round (gray crosses and circles).

How can we optimize parameters, i.e., identify the set of parameters that maximizes the likelihood (or minimizes the SSE)? Figure 3 shows a schematic of likelihood as a function of the parameter value. For simplicity, the parameter is assumed to change its value in one-dimensional space, although the parameter space is usually multi-dimensional in real cases. In the following sections, we introduce some procedures that can be used to identify the set of parameters that maximizes the likelihood. Minimization of SSE can be accomplished with similar procedures.

Optimization procedures can roughly be classified into two categories: gradient and sampling approaches. Gradient approaches search for the (local) maximum of a likelihood based on information from the local gradient, whereas sampling approaches examine numerous sets of parameters and select the sets that attain high likelihood. Gradient approaches can efficiently reach a (local) maximum with small computational cost, although they are inefficient for identifying the global maximum if there are multiple local maxima. In contrast, sampling approaches can detect multiple local maxima, if they exist, but require a massive computational cost.

### Gradient approach

The gradient approach is based on a deterministic method of identifying maximum or minimum values of a given function. When the likelihood, *L*, is a continuous function of the parameters **Θ** = {θ_1_, θ_2_, …}, the optimum parameters can be identified by analytical calculation. The solution of the system of partial differential equations ∂*L*/∂θ_*i*_ = 0(*i* = 1, 2, …) is the set of parameters that yields the local maximum of the likelihood (Figure 3A). This procedure can also be used to minimize the SSE in linear regression analyses.

When it is difficult to solve the system of partial differential equations ∂*L*/∂θ_*i*_ = 0 analytically, we must search for the solution numerically, based on the gradient approach, as follows: (1) set an appropriate initial parameter value **Θ** = **Θ**_0_; (2) compute the gradient of the likelihood for the initial value, i.e., ∂*L*/∂**Θ**|_**Θ** = **Θ**_0__; (3) update **Θ** in the direction of increase of the gradient to increase the likelihood *L*; and (4) iterate (1) through (3) until the gradient converges with zero. We can directly reach one of the (local) maxima of *L* using this deterministic method (Figure 3B). This procedure is used in several areas of biological research, for estimation of values for bending elasticity during cytokinesis (Koyama et al., 2012), transcriptional parameters, and chemotaxis parameters (see later sections).

The gradient approach often leads not to the global maximum but to a local maximum when the likelihood is multimodal, i.e., multiple local maxima exist. To overcome this disadvantage, stochastic procedures are often adopted so that parameters can exit a local maximum by permitting the current searching point to move down the gradient with some probability (Figure 3C). As an example of a biological application of the gradient approach, one of the stochastic methods, the Metropolis algorithm, has been utilized in combination with a simulated annealing method to predict the positions of nucleosomes on the genome (NucPosSimulator, Schöpflin et al., 2013).

### Sampling approach

In principle, if we examined all sets of possible parameters, we could determine the entire form of a given likelihood and, thus, the parameters that yield the maximum likelihood. However, this strategy is not realistic in most cases. Instead, we sample a number of parameter sets and evaluate the likelihood for each set. As the number of samples increases, the parameter set that yields the largest likelihood approaches the optimum one. Roughly speaking, there are two ways to sample parameter sets; one is “grid sampling,” in which a sample is obtained at each parameter grid, at regular intervals (Farhadifar et al., 2007) (Figure 3D), and another is “random sampling,” in which samples are randomly obtained (Bergstra and Bengio, 2012) (Figure 3E). A typical sampling approach does not often work well due to “the curse of dimensionality,” which means that the enormous number of samples required for sufficient coverage of the high-dimensional space are impossible to process. The following two strategies can be used to overcome this problem. The first strategy is importance sampling (Section Importance Sampling), in which parameter space with higher likelihood will be searched recursively, to obtain as many samples as possible from a key area. The second strategy is to narrow the parameter space using prior information. We can statistically incorporate our prior guess using Bayes' theorem (Section Obtaining Posterior PDFs Using a Sampling Approach). In cell and developmental biology, we often have *a priori* information on the order of magnitude of parameter values.

### Importance sampling

Since parameters near the optimum parameters should have high likelihood, we can efficiently search the optimum parameters by focusing the investigation on parameter sets with high likelihood. In “importance sampling” (Figure 3F), after an initial round of grid or random sampling, we repeat the sampling, with greater intensity, near the samples with high likelihood.

An example that utilizes the importance sampling technique is the particle filter (PF), which is often applied to estimate a posterior distribution and/or parameters by means of a number of realizations called “particles.” Genetic algorithms (GAs) (Mitchell, 1998) are similar to PFs in that they both select important samples in accordance with likelihood (or other indices). However, GAs are not usually categorized as importance sampling methods because the outcomes are not guaranteed to converge to the target distribution function, due to stochastic events (“mutation” or “crossover”) unrelated to the likelihood.

## Bayesian inference of parameter distribution

The above-mentioned sampling approaches enable us to determine not only the parameter set that yields the maximum likelihood but also the likelihood of all samples. Utilizing this information, we can estimate, in principle, the entire form of the likelihood function within the parameter space. Calculation of the likelihood function provides important information on the inevitable measurement noise in biological experiments and the uncertainty of given stochastic models.

Unlike a straightforward approach to obtain the likelihood function using all possible sets of parameters, which would be unrealistic, a Bayesian approach provides a powerful and realistic methodology to estimate target PDFs as posterior distributions. In real data analyses or modeling, we often have prior information about parameters, e.g., a realistic range of parameters obtained through experimentation. Bayesian inference methods make use of prior information in order to limit the parameter space to be searched.

The outcome of the inference is a “posterior PDF,” *p*(**Θ**|***Y***), which indicates how probable a parameter set **Θ** = {θ_1_, θ_2_, …} is when ***Y***, usually an experimental observation, is given. In contrast, the prior PDF p(**Θ**) indicates how probable **Θ** is without knowing ***Y***. The prior PDF reflects our initial guess of the parameter value. For example, if one supposes that a parameter must be within the range from 1 to 100 but has no additional information, a uniform distribution on the interval from 1 to 100 is the appropriate prior PDF. According to Bayes' theorem, the posterior PDF is proportional to the product of the prior PDF and the likelihood, which is formulated as p(**Θ**|***Y***) = p(***Y***|**Θ**) × p(**Θ**)/p(***Y***) (Lee, 2012). Here, p(***Y***|**Θ**) is the likelihood, which expresses how probable ***Y*** is when the parameter **Θ** is given, and p(***Y***) is a PDF related to the observed data, ***Y***, which is constant. It should be noted that the likelihood is not a probability distribution in the sense that its integral does not necessarily equal one (Bishop, 2006). Combining Bayesian inference with the sampling approach (Section Obtaining Posterior PDFs Using a Sampling Approach) or the gradient approach (Section Obtaining Posterior PDFs Using a Gradient Approach) enables us to obtain both likelihood and posterior PDFs.

### Obtaining posterior PDFs using a sampling approach

**Figure 4:**
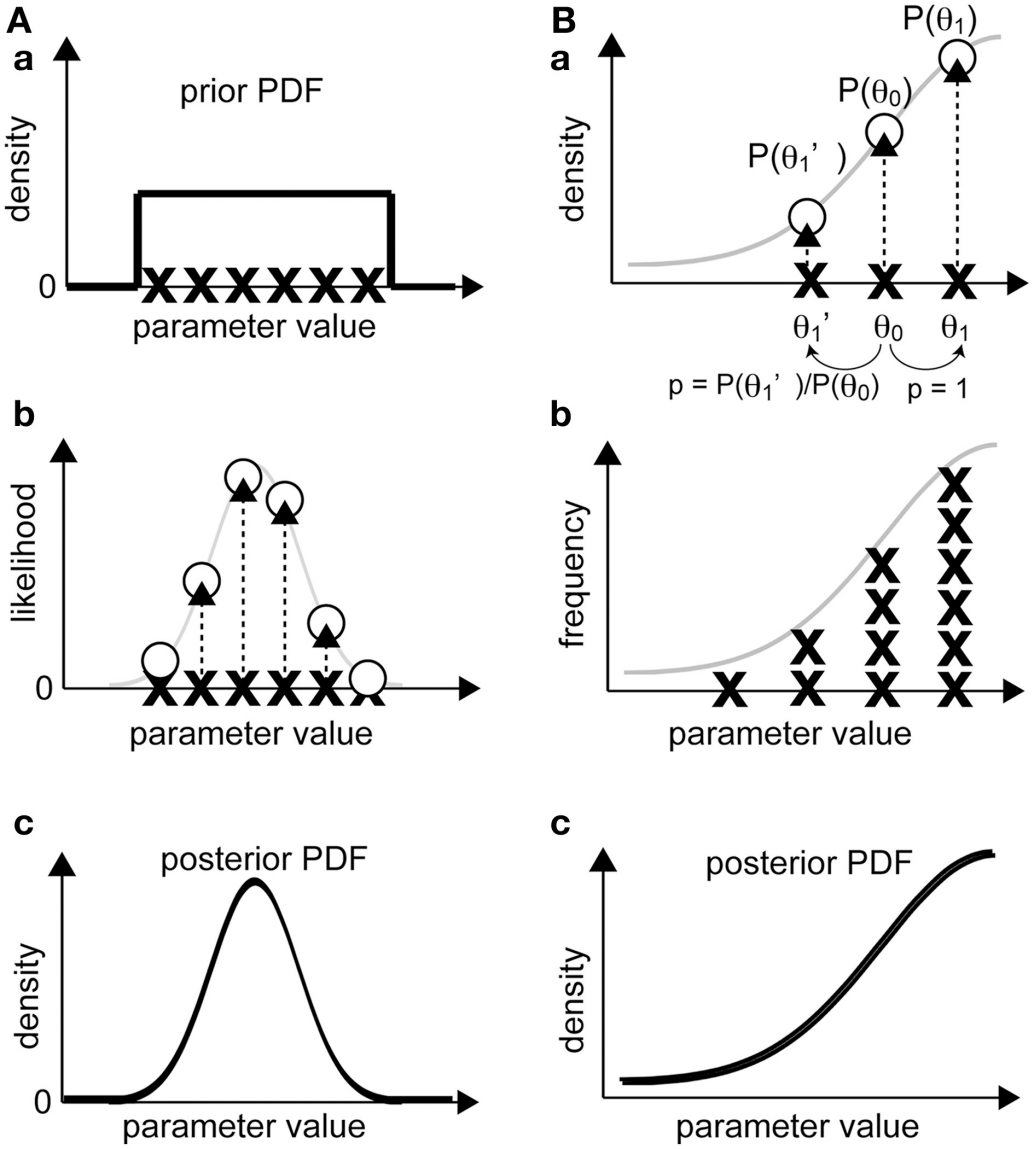
**Bayesian inference of parameter distribution**. **(A)** In the sampling approach, the likelihood of observing experimental data for a realization is calculated (a, b). Then, the non-normalized posterior PDF is calculated by interpolating the likelihood values in the parameter space between the realizations (c). **(B)** In the gradient approach, a realization (e.g., θ_0_) is randomly shifted to a neighboring realization (θ_1_ or θ_1_'). If the product of the likelihood and the prior probability of the new realization is greater than that of the original, the old realization will be replaced by the new realization and sampled. If the product for the new realization is smaller, the realization will be replaced by the new set, and the probability of this new set will be given as the ratio of the products of the new and the old realizations (otherwise, the original realization will not be replaced), and the realization will be sampled (a). After repeating the procedure multiple times (b), the distribution of the sampled realizations is considered proportional to the posterior PDF (c).

In this approach (Figure 4A), we sample a number of sets of parameter values, which are termed as “realizations,” according to the prior PDF [Figure 4A(a)]. Then, we calculate the likelihood of each realization by substituting it into our model [Figure 4A(b)]. According to Bayes' theorem, the unnormalized posterior PDF, which is proportional to the normalized one, is obtained as a product of the likelihood and the prior PDF for each realization. Since we sampled from the prior PDF, the unnormalized posterior PDF is the likelihood at the sampling points whose deviation already reflects prior effects [Figure 4A(c)]. The normalized posterior PDF can be calculated by dividing the unnormalized posterior PDF by p(***Y***), but this calculation requires a complex numerical integration. Without such normalization, the form of the function for the normalized and unnormalized posterior PDFs are identical, and thus the optimum parameter set can be obtained from the unnormalized one because p(***Y***) is constant. Therefore, calculation of an unnormalized posterior PDF is usually sufficient for our purposes. The parameter set at the mode of the unnormalized posterior PDF, i.e., the parameter set that attains the posterior PDF maximum, is called the maximum-a-posteriori (MAP) estimate.

PF, or sequential Monte Carlo, is a filtering method that is used to sequentially estimate, using importance sampling, posterior PDFs along with continuous input of observation data. Sets of parameters (“particles”) with a high likelihood will proliferate (or will be “resampled,” allowing duplication) (Figure 3F). Unlike GA, which focuses on finding the optimum set, PF enables us to estimate the likelihood and the posterior PDF. To avoid the problem of “degeneration,” which the plain PF often faces, some advanced PF methods, such as merging PF (Nakano et al., 2007), have been proposed. A real application of PF to estimation of parameters can be found in studies on transcriptional regulation of the circadian clock (Nakamura et al., 2009).

### Obtaining posterior PDFs using a gradient approach

In this subsection, we explain the procedure of the Metropolis algorithm (Figure 4B), which applies when the proposal density function that nominates the next candidate realization is symmetric (Gilks et al., 1995; Robert and Casella, 2010). Unlike the above-mentioned sampling approach, in which we calculate the likelihood of multiple and independent realizations, this method starts with a single realization. To obtain a posterior PDF as the target distribution, the sampling procedure is as follows. First, we calculate the value of the posterior PDF related to the initial realization (*P_former_*), which is given by the product of the likelihood and the prior PDF. Next, the proposal density function randomly generates a new candidate realization, and we calculate the value of the posterior PDF (*P_latter_*). If *P_latter_* > *P_former_*, the candidate realization is accepted as a new realization of the posterior PDF. The key step in the Metropolis algorithm is that even if *P_former_* > *P_latter_*, the candidate realization is accepted with the probability of *P_latter_*/*P_former_* [Figure 4B(a)]. When a candidate is rejected, the former realization remains as the current realization. This sampling process is repeated until we obtain a sufficient number of realizations [Figure 4B(b)]. The process that allows a realization to move in the direction of decreasing posterior PDF provides a way to exit local maxima of posterior PDF. The distribution of the sampled realizations approximates the unnormalized posterior PDF [Figure 4B(c)], from which we can calculate the MAP, i.e., the optimum set of parameters that maximizes the posterior PDF.

The procedures for obtaining a posterior PDF using sampling methods based on the Markov process are generally referred to as Markov chain Monte Carlo methods. In this class, in addition to the Metropolis algorithm explained above, Gibbs sampling and Hamiltonian Monte Carlo algorithms are popular (Bishop, 2006). Approximate Bayesian computation (ABC) is another sampling approach that can be used to obtain a posterior PDF (Beaumont et al., 2002). The most remarkable feature of ABC is that instead of likelihood, any index in data space, such as SSE, can be used to determine acceptance/rejection of candidate realizations of parameters. Although it does not employ likelihood, ABC enables us to obtain samples from a target posterior PDF; the convergence speed strongly depends on the definition of the index in data space. This procedure has been used for estimation of the parameters for microtubule dynamics in a plant cell (Nakaoka et al., 2015). The estimated parameters were consistent with the values measured in independent experiments.

## Examples of cellular parameter optimization

### Transcriptional regulation

The initiation and elongation of gene transcription consist of multiple processes involving various regulatory proteins. Darzacq et al. constructed a simple model of transcriptional regulation consisting of three first-order ordinary differential equations describing promoter assembly, transcriptional initiation, and elongation (Darzacq et al., 2007). The six parameters in this model were optimized to fit the experimental results obtained through FRAP analyses of RNA polymerase II in cultured cells by minimizing the SSE. The optimization was conducted using the software SAAM II (The Epsilon Group, Charlottesville, USA).

More recently, Stasevich et al. quantified the accumulation of RNA polymerase II, discriminating between the initiation form (phosphorylated at Ser5 at its C-terminal domain, CTD) and the elongation form (phosphorylated at Ser2) using FabLEM (antibody fragment-based live endogenous modification labeling) technology (Stasevich et al., 2014). Combined with the results of the FRAP assays, the authors were able to narrow the optimum parameters for transcription kinetics. Minimization of the SSE was performed using the software Mathematica (Wolfram, Champaign, USA). Through these analyses, the authors succeeded in quantitatively and precisely characterizing the effect of histone acetylation on transcriptional regulation.

### Bacterial chemotaxis

**Figure 5:**
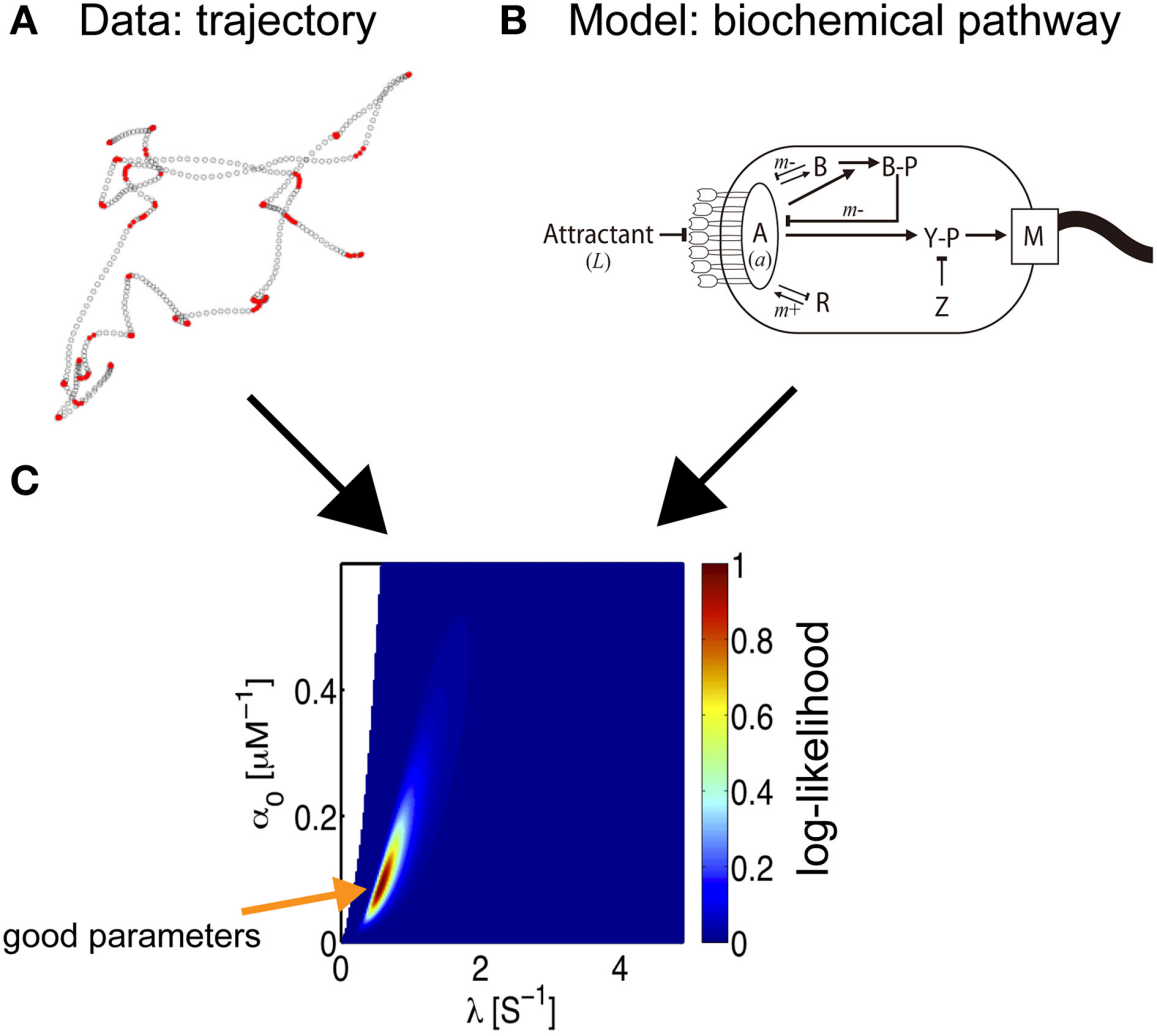
**Parameter estimation of bacterial behavior**. The inference of biochemical parameters in the bacterial chemotaxis pathway from trajectories (Masson et al., 2012). **(A)** Bacteria swimming in a microfluidic device in the presence of a stable, linear chemical gradient (here, Me-Asp) are tracked. According to the current linear speed and angular velocity, a state is associated with the bacterial motion, run (empty circles) or tumble (red circles). The coordinate along the gradient is proportional to the concentration experienced by the bacterium. The time-series of states and concentrations are the input data for the inference process. **(B)** Starting from the full biochemical network, an approximate description of moderate gradient intensity yields an inhomogeneous Poisson model for bacterial states, where the transition rates are related to the kinetic parameters of the model (Celani et al., 2011). An exact expression for the log-likelihood can then be written. **(C)** A 2D section of the likelihood landscape. The abscissa indicates the time-scale of the response, which is governed by the methylation process. The ordinate is the amplitude of the response, which mainly depends on the receptor kinetics. The maximum likelihood estimate indicates the optimum choice of parameters for the model.

The impulse response of bacteria has been estimated from bacterial chemotaxis trajectories, using inference methods (Masson et al., 2012). The model organism *Escherichia coli* senses the environmental concentration of chemicals and uses that information to regulate the rotation of flagellar motors and thus orient its trajectories of motion (Berg, 2004). Information on the chemical concentration sensed by the receptors is relayed via the kinase CheA, and the activity of this molecule is reduced by receptor binding. The second messenger in the chemotaxis pathway is the protein CheY. Its phosphorylated form, CheYp, binds to the flagellar motors and increases their rate of switching from counterclockwise rotation, corresponding to run phases, to clockwise rotation, thereby destabilizing the flagellar bundles that induce tumbling. Other important components of the pathway include the scaffold protein CheW, the phosphatase CheZ, the methyltransferase CheR, and the methylesterase CheB; the latter two are responsible for feedback from the receptors and the resulting adaptation (see Figure 5 and Vladimirov and Sourjik, 2009 for a recent review).

Is it possible to reconstruct the kinetics of biochemical interactions from an analysis of bacterial trajectories? In other words, can we infer the molecular pathways from paths in physical space? The task was greatly simplified by the fact that the model that describes the observations was known in advance, based on previous independent experimental assays and modeling efforts (reviewed in Celani et al., 2011). The goal was then reduced to the identification of the appropriate parameters. Furthermore, for the problem at hand, under physiological conditions, the response is linear. This convenient property allowed bacterial movement to be described as a two-state, inhomogeneous Poisson process, and closed-form expressions for the likelihood of a trajectory can be obtained. Additionally, in view of the compactness of the pathway, only three parameters are relevant: the intensity of the response (*α*_0_), its duration (*α*_1_), and the degree of adaptation (λ), and the impulse response can be described as a function of time (*t*) by *K*(*t*) = e^−^*^λt^* × (*α*_0_ − *λα*_1_*t*). These quantities are directly related to various molecular parameters, such as receptor affinities, protein copy numbers, and (de-)phosphorylation and (de-)methylation rates. The small number of parameters then allows for an exhaustive exploration of parameter space and a straightforward derivation of the best parameter set for the model.

Remarkably, when the trajectory of a single bacterium is tracked for a sufficiently long time, it is possible to infer the values of molecular parameters for that individual, allowing us to probe variations within a given isogenic population (Masson et al., 2012). To maximize the likelihood, optimization was performed using two types of gradient methods, a variable metric method and a simplex algorithm combined with a conjugate gradient method, and the MAP solution was calculated. Both methods yielded the same results, within acceptable statistical uncertainty. Another notable advantage of this inference technique is its non-invasive nature; swimming bacteria are observed under the microscope and are not disturbed by the observation.

### Morphogenesis of tissues and organs

Mechanical forces are critical for the morphogenesis of tissues and organs. However, such forces are difficult to measure. For example, if an object is not moving, we cannot tell whether a small force is acting on the object or strong forces are acting on the object but are balanced out. One way to estimate such forces is to ablate a part of a tissue/organ and measure the speed and direction in which the lesion spreads. This method is invasive and cannot be repeated for a given sample. Recent studies developed methods to infer a stress or deformation map during morphogenesis. To infer stress distribution in epithelial tissues (Chiou et al., 2012; Ishihara and Sugimura, 2012), the authors first constructed physical models assuming that the force balance involving tensions at cell contact surfaces and pressures of cells determines shapes of epithelial cells. The methods search for model parameters that reproduce the cell shapes in the tissues quantified from microscope images. In the method proposed by Chiou et al. (2012), the tension and pressure of constituent cells were estimated analytically. In comparison, the method proposed by Ishihara and Sugimura (2012) reduces the number of parameters to be optimized to two, i.e., the variance of tension and the variance of observation/system errors. These parameters were optimized analytically or numerically through a gradient method. The authors were able to demonstrate the validity of the estimation by experimentally measuring the tension using laser ablation (Ishihara and Sugimura, 2012). The method was further utilized to demonstrate the importance of extrinsic anisotropy in mechanical forces for *Drosophila* wing development (Sugimura and Ishihara, 2013). A similar method was developed to create a deformation map of a whole organ during chick limb development (Morishita and Suzuki, 2014). This map precisely describes the type of deformation and its temporal regulation during organ morphogenesis.

### Cell cycle regulation

The molecules that drive cell cycle progression and their relationships are well-studied. Detailed numerical models consisting of a number of molecules accurately reflect current experimental knowledge (Borisuk and Tyson, 1998; Tsai et al., 2008). Kondo et al. attempted to simplify the detailed models to identify “low-dimensional” models that sufficiently reproduce the observations of the detailed models (Kondo et al., 2013). The authors first constructed models with two dimensions (considering only active Cdc2 and cyclin) and various polynomial orders then optimized the parameters using a PF method. By calculating the AIC and BIC of the models, the authors concluded that the model with a third-order polynomial sufficiently reproduces characteristic behaviors of the cell cycle models.

## Perspective: from explanation to prediction

Data-driven science is gaining popularity in most scientific fields. With the rapid development of information technology, scientists can collect “big data” in their field and develop new methods for analysis. Use of such methodologies in other fields will provide clues regarding biological data analysis. For example, data assimilation (DA) is a fundamental computing technique used to predict future states by an integration of numerical simulation models and time-series data, using Bayesian statistics. DA has been used in weather forecasting and in predicting the status of the Earth's interior that may trigger a large earthquake (Nagao et al., 2013). It has been applied to dynamic biological systems such as circadian rhythms (Nakamura et al., 2009). The method is also important in control theory for estimating the internal state of interest. Lillacci and Khammash applied an extended Kalman filter for parameter estimation in non-linear biological systems, including the heat shock response in *E. coli* (Lillacci and Khammash, 2010). An accurate prediction of the (unknown) future is not required in the field of experimental biology, which focuses on the explanation of experimental results. Importantly, the method enables us to conduct “on-line modeling,” in which a model is improved simultaneously with data acquisition. Such on-line modeling may be useful for the imaging of a moving object by controlling the field of view of the microscope with predictive information with respect to movement. In general, the concepts and techniques used in cutting-edge statistics should be applicable to the field of experimental biology. With this in mind, we anticipate that a collaborative, trans-disciplinary approach will become more and more important in quantitative biology.

### Conflict of interest statement

The authors declare that the research was conducted in the absence of any commercial or financial relationships that could be construed as a potential conflict of interest.

## References

[B1] Akaike H. (1974). A new look at the statistical model identification. IEEE Trans. Automat. Contr. 19, 716–723 10.1109/TAC.1974.1100705

[B2] Axelrod D. Koppel D. E. Schlessinger J. Elson E. Webb W. W. (1976). Mobility measurement by analysis of fluorescence photobleaching recovery kinetics. Biophys. J. 16, 1055–1069. 10.1016/S0006-3495(76)85755-4 786399 PMC1334945

[B3] Beaumont M. A. Zhang W. Balding D. J. (2002). Approximate Bayesian computation in population genetics. Genetics 162, 2025–2035. 12524368 10.1093/genetics/162.4.2025 PMC1462356

[B4] Berg H. C. (1993). Random Walks in Biology. Princeton, NJ: Princeton University Press.

[B5] Berg H. C. (2004). E. coli, in Motion, ed Berg H. C. (New York, NY: Springer Science and Business Media), 5–16.

[B6] Bergstra J. Bengio Y. (2012). Random search for hyper-parameter optimization. J. Mac. Learn. Res. 13, 281–305.

[B7] Bishop C. M. (2006). Pattern Recognition and Machine Learning. New York, NY: Springer Verlag.

[B8] Borisuk M. Tyson J. (1998). Bifurcation analysis of a model of mitotic control in frog eggs. J. Theor. Biol. 195, 69–85. 10.1006/jtbi.1998.0781 9802951

[B9] Bremer M. Doerge R. W. (2010). Statistics at the Bench: A Step-by-step Handbook for Biologists. Cold Spring Harbor, NY: Cold Spring Harbor Laboratory Press.

[B10] Celani A. Shimizu T. S. Vergassola M. (2011). Molecular and functional aspects of bacterial chemotaxis. J. Stat. Phys. 144, 219–240 10.1007/s10955-011-0251-6

[B11] Chiou K. K. Hufnagel L. Shraiman B. I. (2012). Mechanical stress inference for two dimensional cell arrays. PLoS Comp. Biol. 8:e1002512. 10.1371/journal.pcbi.1002512 22615550 PMC3355066

[B12] Darzacq X. Shav-Tal Y. de Turris V. Brody Y. Shenoy S. M. Phair R. D.. (2007). *In vivo* dynamics of RNA polymerase II transcription. Nat. Struct. Mol. Biol. 14, 796–806. 10.1038/nsmb1280 17676063 PMC4942130

[B13] Farhadifar R. Röper J.-C. Aigouy B. Eaton S. Jülicher F. (2007). The influence of cell mechanics, cell-cell interactions, and proliferation on epithelial packing. Curr. Biol. 17, 2095–2104. 10.1016/j.cub.2007.11.049 18082406

[B14] Gilks W. R. Richardson S. Spiegelhalter D. (1995). Markov Chain Monte Carlo in Practice. London: CRC Press.

[B15] Hara Y. Kimura A. (2009). Cell-size-dependent spindle elongation in the *Caenorhabditis elegans* early embryo. Curr. Biol. 19, 1549–1554. 10.1016/j.cub.2009.07.050 19682904

[B16] Hara Y. Kimura A. (2013). An allometric relationship between mitotic spindle width, spindle length, and ploidy in *Caenorhabditis elegans* embryos. Mol. Biol. Cell 24, 1411–1419 10.1091/mbc.E12-07-0528 23468523 PMC3639052

[B17] Hastie T. Tibshirani R. Friedman J. (2009). The Elements of Statistical Learning. New York, NY: Springer Science and Business Media 10.1007/978-0-387-84858-7

[B18] Ishihara S. Sugimura K. (2012). Bayesian inference of force dynamics during morphogenesis. J. Theor. Biol. 313, 201–211. 10.1016/j.jtbi.2012.08.017 22939902

[B19] Jaqaman K. Danuser G. (2006). Linking data to models: data regression. Nat. Rev. Mol. Cell Biol. 7, 813–819. 10.1038/nrm2030 17006434

[B20] Kitagawa G. (2010). Introduction to Time Series Modeling. Boca Raton, FL: CRC Press 10.1201/9781584889229

[B21] Kondo Y. Kaneko K. Ishihara S. (2013). Identifying dynamical systems with bifurcations from noisy partial observation. Phys. Rev. E 87:042716. 10.1103/PhysRevE.87.042716 23679458

[B22] Koyama H. Umeda T. Nakamura K. Higuchi T. Kimura A. (2012). A high-resolution shape fitting and simulation demonstrated equatorial cell surface softening during cytokinesis and its promotive role in cytokinesis. PLoS ONE 7:e31607 10.1371/journal.pone.0031607 22359606 PMC3281004

[B23] Lee P. M. (2012). Bayesian Statistics. Chichester: John Wiley and Sons.

[B24] Lillacci G. Khammash M. (2010). Parameter estimation and model selection in computational biology. PLoS Comp. Biol. 6:e1000696. 10.1371/journal.pcbi.1000696 20221262 PMC2832681

[B25] Masson J.-B. Voisinne G. Wong-Ng J. Celani A. Vergassola M. (2012). Noninvasive inference of the molecular chemotactic response using bacterial trajectories. Proc. Natl. Acad. Sci. U.S.A. 109, 1802–1807. 10.1073/pnas.1116772109 22307649 PMC3277171

[B26] Mitchell M. (1998). An Introduction to Genetic Algorithms. Cambridge: MIT Press.

[B27] Morishita Y. Suzuki T. (2014). Bayesian inference of whole-organ deformation dynamics from limited space-time point data. J. Theor. Biol. 357, 74–85. 10.1016/j.jtbi.2014.04.027 24810841

[B28] Nagao H. Higuchi T. Miura S. Inazu D. (2013). Time-series modeling of tide gauge records for monitoring of the crustal activities related to oceanic trench earthquakes around Japan. Comput. J. 56, 355–364 10.1093/comjnl/bxs139

[B29] Nakaoka Y. Kimura A. Tani T. Goshima G. (2015). Cytoplasmic nucleation and atypical branching nucleation generate endoplasmic microtubules in Physcomitrella patens. Plant Cell 27, 228–242 10.1105/tpc.114.134817 25616870 PMC4330588

[B30] Nakamura K. Yoshida R. Nagasaki M. Miyano S. Higuchi T. (2009). Parameter estimation of *in silico* biological pathways with particle filtering towards a petascale computing. Pac. Symp. Biocomput. 14, 227–238. 10.1142/9789812836939_0022 19209704

[B31] Nakano S. Ueno G. Higuchi T. (2007). Merging particle filter for sequential data assimilation. Nonlin. Processes Geophys. 14, 395–408 10.5194/npg-14-395-2007

[B32] Robert C. Casella G. (2010). Monte Carlo Statistical Methods. New York, NY: Springer.

[B33] Saxton M. J. Jacobson K. (1997). Single-particle tracking: applications to membrane dynamics. Annu. Rev. Biophys. Biomol. Struct. 26, 373–399. 10.1146/annurev.biophys.26.1.373 9241424

[B34] Schöpflin R. Teif V. B. Müller O. Weinberg C. Rippe K. Wedemann G. (2013). Modeling nucleosome position distributions from experimental nucleosome positioning maps. Bioinformatics 29, 2380–2386. 10.1093/bioinformatics/btt404 23846748

[B35] Stasevich T. J. Hayashi-Takanaka Y. Sato Y. Maehara K. Ohkawa Y. Sakata-Sogawa K.. (2014). Regulation of RNA polymerase II activation by histone acetylation in single living cells. Nature 516, 272–275. 10.1038/nature13714 25252976

[B36] Sugimura K. Ishihara S. (2013). The mechanical anisotropy in a tissue promotes ordering in hexagonal cell packing. Development 140, 4091–4101. 10.1242/dev.094060 24046322

[B37] Tsai T. Y.-C. Choi Y. S. Ma W. Pomerening J. R. Tang C. Ferrell J. E. (2008). Robust, tunable biological oscillations from interlinked positive and negative feedback loops. Science 321, 126–129. 10.1126/science.1156951 18599789 PMC2728800

[B38] Vladimirov N. Sourjik V. (2009). Chemotaxis: how bacteria use memory. Biol. Chem. 390, 1097–1104. 10.1515/BC.2009.130 19747082

